# Evaluating barbed sutures: A porcine biomechanical comparison of Z-plasty and turndown flap according to Silfverskiöld

**DOI:** 10.1016/j.jor.2025.03.003

**Published:** 2025-03-10

**Authors:** Henry V. Bürger, Jahnke Alexander, Harz Torben, Carlos A. Fonseca-Ulloa, Markus Rickert, Dirk Stolz

**Affiliations:** a Laboratory of Biomechanics, Department of Orthopaedics and Orthopaedic Surgery, Justus-Liebig-Universität Gießen, Klinikstraße 29, 35392, Gießen, Germany; b Department of Orthopaedics and Orthopaedic Surgery, Universitätsklinik Gießen Marburg (UKGM), Klinikstraße 33, 35392, Gießen, Germany

**Keywords:** Barbed suture, Tendon, Stratafix, Biomechanics, Z-plasty, Turndown flap, Silfverskiöld

## Abstract

**Introduction:**

It is not possible to add an infinite amount of suture material to tendon plasties and repairs in vivo. Each additional knot can reduce the tensile strength by up to 50 %. Therefore, barbed sutures, as a knotless suturing system, should be investigated as a potential alternative to traditional sutures to minimize knot-related weakening.

**Material and methods:**

Superficial porcine flexors were randomized into five groups. A non-contact measurement was utilized. The Z-plasty and the turndown flap according to Silfverskiöld were used. The Stratafix barbed knotless suture was compared to regular smooth polydioxanone. The biomechanical protocol included a creep test, a cyclic test, and a tear-off test.

**Results:**

The Z-plasty with Stratafix showed significantly improved maximum force compared to the Z-plasty with Polydioxanon (PDS 108.5 ± 22.2N, Stratafix 142.3 ± 23.5N, p < .01). The Z-plasty was significantly superior to the turndown flap in maximum Force (turndownflap with Stratafix 52.4 ± 14.6N, Z-plasty with Stratafix 108.5 ± 22.2N, p < .001).

**Conclusion:**

The Stratafix barbed suture can significantly improve the Z-plasty in maximum tension by up to 32 % when compared to regular PDS. To formulate a more precise indication, biological factors must be further investigated.

## Highlights

• 32 % improvement in maximum force without the use of additional suture material.
• Comparative evaluation of rupture mechanism with barbed sutures.
• Reduced risk of complications associated with suture knots.
• A potentially more effective method of performing Z-plasty in Achilles tendons.

## 1 Introduction

### 1.1 Barbed sutures and smooth monofilament PDS

Barbed sutures, such as the Stratafix, differ in design and application from smooth monofilament sutures. They are both made from polydioaxonone (PDS), a synthetic, absorbable material.

Barbed sutures come with small barbs of varying sizes that evenly distribute tension across the tissue. They frequently have a suture anchor for secure attachment, eliminating the need for knots.^1^ This knotless design can shorten operation times, reducing suture slippage and pressure points^2^-.^4^ They can cause tissue damage if placed incorrectly or repositioned and they can only be slightly adjusted once inserting.^2^ Tissues that require more flexibility due to dynamic stress may be better suited to traditional smooth sutures.^5^ Barbed sutures can only be placed in running matter, and suture breakage then jeopardizes the entire repair.^2^

Regular PDS is a smooth monofilament suture. It offers great tensile strength but requires knot-tying for secure placement without supplementary mechanisms such as barbs or suture anchors.^6^^,^^7^ Each stitch can be placed individually, offering stability and flexibility. The tension can be adjusted separately, which is beneficial for uneven tissue.^1^^,^^8^ Tying of surgical knots elongates and weakens the material. It brings variety and human error possibilities.^3^^,^^9^

### 1.2 The Achilles tendon rupture

The Achilles tendon rupture (ATR) is the most common rupture, with an incidence rate of 12–20 cases per 100.000 patients annually, peaking between the ages of 30 and 50. Chronic, degenerative ruptures peak at the age of 60 ^10^. Due to the rising incidence of Achilles tendon ruptures, particularly in active individuals aged 30–50, and due to demographic changes, effective repair techniques are increasingly important..^10^^,^^11^ Male patients have a 5-6-time increased risk.^12^^,^^13^ Additionally, re-ruptures caused by tendinopathy must be considered.^11^^,^^14^ The treatment depends on the type of rupture, localization, and size of the defect. Both conservative and surgical procedures can be used.^15^ Conservative procedures have an elevated risk of re-rupture.^16^ Optimal surgical routine is critical since prolonged immobilization increases the risk of pulmonary embolism and other complications.^17^ The techniques of end-to-end anastomosis, according to Bunnel, Krackow, or Kirschmayr-Kessler, have proven successful in various studies and in daily clinical practice. In cases of chronic or neglected ruptures, reconstruction techniques like turndown flaps or additional synthetic materials might be necessary.^18^^,^^19^

### 1.3 Suture techniques

The turndown flap, according to Silfverskiöld, was specifically designed for chronic or severe Achilles tendon ruptures. This technique's principle involves “folding down” a segment of the gastrocnemius or soleus tendon to bridge the defect and provide structural support.^20^^,^^21^ This flap is secured to the distal tendon using sutures (see Fig. 2 for schematics). While the primary goal of this technique is to repair tendon ruptures by filling a defect with additional tendon tissue, its underlying principle also suggests potential applicability for tendon lengthening.

**Fig. 1:**
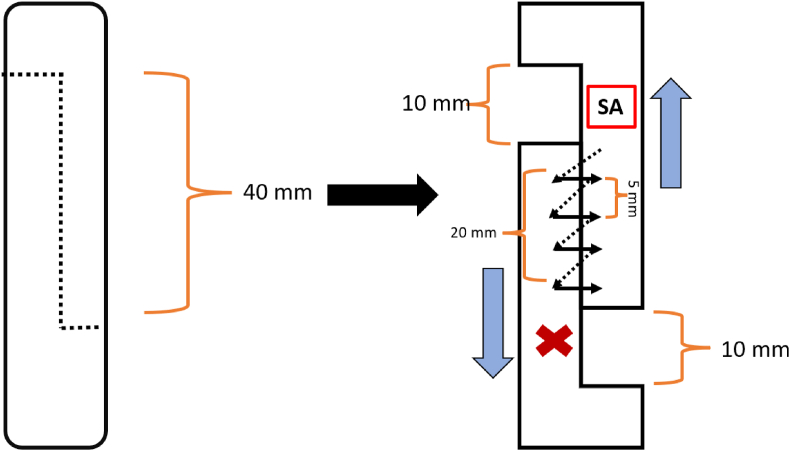
Image a, no color **Caption:** Z-plasty scheme *Description*. The red “SA” indicates the suture anchor position for Stratafix and the starting point for PDS. The red “x” marks the endpoint, where a cross-stitch is applied for Stratafix and a regular surgical knot for PDS. The arrows represent the four stitches placed in a simple running fashion.

**Fig. 2:**
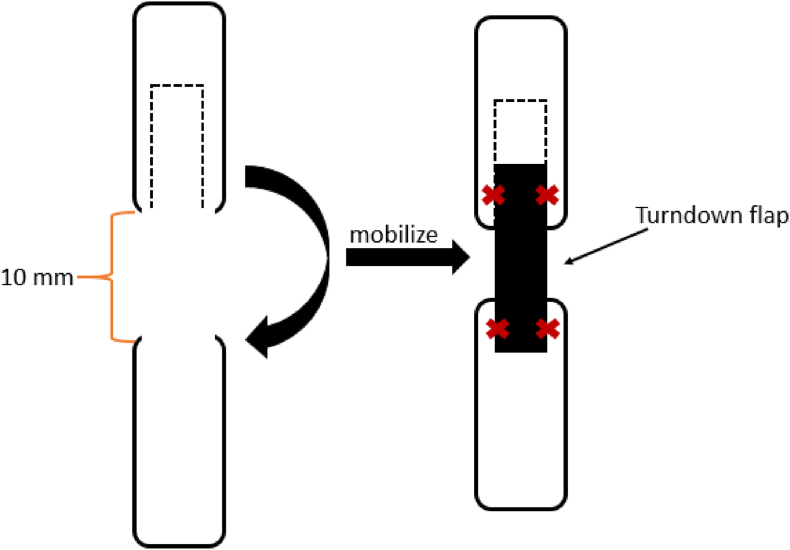
Image b, no color: **Caption:** Scheme of turndown flap according to Silfverskjöld.

However, tendon lengthening is more frequently accomplished with methods like the Z-plasty, which are designed especially for controlled elongation in different tissues. Generally the Z-plasty is a surgical technique used to enhance the mobility and functionality of tissue.^20^ The technique can be adapted for various deformities and contractures. It can help to prevent long-term dysfunction and improve overall rehabilitation outcomes by promoting better tendon excursion and reducing the risk of re-adhesion.^20^^,^^21^ The procedure involves making two diagonal incisions that form a “Z" shape, allowing the surgeon to realign tissue, reduce tension, and lengthen contracted or tight areas. For Achilles tendons, this technique is used to lengthen the tendon^22^^,^^23^ (see Fig. 1).

### 1.4 Hypothesis

Numerous in vitro biomechanical studies have added synthetic material to tendon repairs to achieve greater loads. In vivo endless augmentation is not possible due to technical limitations and reduced movement within the tendon sheath, additionally each knot may reduce tensile strength by as much as 50 percent (%)^24^ Therefore, the primary objective of this study is to test whether the biomechanical properties of barbed sutures are favorable for the techniques Z-plasty and turndown flap according to Silfverskiöld. The small barbs and knotless design may result in a more even distribution of force and greater primary stability. Additionally, we explore whether the turndown flap technique, originally designed for tendon defects, could also serve as a method for controlled tendon lengthening.

## 2 Material and Methods

### 2.1 Specimen preparation

Due to similarities to the human Achilles tendon, the porcine flexor tendons of the common domestic pig were used^25^-.^28^ Tendons with macroscopic abnormalities were excluded. The tendons were deep frozen in aluminum foil at −20 °C (°C). An incubator (Brutschrank IN110, Memmert GmbH + Co.KG, Büchenbach) defrosted the tendons in a 37 °C, 0.9 % sodium chloride solution before testing.^27^

### 2.2 Study size

Five groups of ten were recruited for this study to compare different clinical applications. One group served as a control with no defect and no suture material, providing a baseline for the experimental groups. The remaining four groups compared two suture techniques, each tested with two different materials. This setup allowed for the evaluation of one technique primarily used for ruptures and another for tendon lengthening, analyzing how each variable impacts specific biomechanical properties.

### 2.3 Measurement

The cross-section was determined with laser optics. For this purpose, the tendons were initially uniaxially clamped in a universal testing machine (Inspekt table Blue 20 EDC 222, Hegewald & Peschke Meβ-und Prüftechnik GmbH, Nossen, Germany). We wrapped the proximal and distal ends of the tendons in a 5 × 10 cm (cm) bandage to prevent displacement. The cross-sectional measurement was performed analogously to existing literature using a preload of 10 N (N).^25^^,^^27^ We placed the defect at the thinnest part of the tendon.

### 2.4 Suture technique

The setup utilizes a wooden board with a millimeter scale attached and three screws as anchor points for the elastic bands. They were wrapped around the tendon, ensuring even tension for the 10 mm (mm) elongation.

For the Z-plasty, a 4 cm incision was made along the direction of the tendon's fibers. The tendon ends were displaced against each other until an elongation of 10 mm was achieved. The suture anchor was placed at the exact location the knot would be. Side-to-side anastomosis was performed over 2 cm with 0.5 cm distance between each stitch in simple running fashion. A knot secured the smooth pds and cross stitch the Stratafix *(see Image a for schematic)*.

The turndown flap according to Silfverskiöld was used to augment a modified Kessler technique. About 3 cm from the defect, parallel to the defect and extending half the diameter of the tendon, a 2 cm long flap was dissected *(see Image b)*. This tendon flap “turned down” over the defect and secured with four simple frame sutures.

### 2.5 Suture material

Absorbable smooth monofilament polydioxanone suture size 0, 3.5 metric (PDS II™, Johnson&Johnson Medical Ltd., Livingston, England) and the Stratafix size 0, 3.5 metric were used for this study (Stratafix Symmetric PDS ™ Plus, Johnson&Johnson Medical Ltd.). The suture material was tested in a ZwickRoell Testing Machine (ZwickRoell GmbH&Co.KG Ulm, Germany).

### 2.6 Biomechanical protocol

The protocol included a creep test, cyclic test, and a tear-off test. A mandatory break of 30 min was taken between each of the tests. Every 5 min the tendon was moistened with 0.9 % sodium chloride (NaCl). A preload and afterload phase with 10N over a period of 90 s was performed for each test. The creep test started to build up stress at 5 N per second (N/s) until 30N were reached and were held for 15 min. Forces ranging from 2N to a maximum of 30N for 500 runs at a frequency of 0.4 Hz (Hz) were used for the cyclic test*.* This corresponds to slow walking in the early rehabilitation phase.^25^ The tear-off test was then performed at 300 N/s to test the maximum load until failure, the maximum force, and the maximum stress.

### 2.7 Statistical method

Statistical analysis was performed using SPSS 29 (Windows, IBM, Amronk, NY, USA) and Graphpad Prism version 9 (Windows, GraphPad Software, Inc. San Diego, CA, USA). For the creep test and the cyclic test, the percent creep strain in % was determined and evaluated for each test separately. For the tear-off test the parameters maximum strain in %, maximum force in N and maximum stress in Newton per square millimeter (N/mm^2^) were evaluated. The data was analyzed for normal distribution using the Shapiro-Wilk test at a confidence interval of 95 %. For normally distributed data, a two-factor ANOVA was performed for the variables suture technique and suture material. The Kruskal-Wallis test was used for non-normal distributed data.

## 3 Results

### 3.1 Cross-section measurements

The average cross section for all tendons was 19.7±1 mm. There were no statistically significant cross-sectional differences between the five experimental groups.

### 3.2 Testing the suture material and native tendon

We were able to determine the maximum force of 99.3 ± 11.6 N for the Stratafix suture and 150.5 ± 18 N for the PDS (p < .001). For the native tendon, 1448 ± 322.4N of maximum force and 69.2 ± 13.9 N/mm^2^ maximum tension.

### 3.3 Creep test

No significant differences were observed in the creep test between suture materials for the turndown flap (PDS 43.6 ± 28.1 %; Stratafix 40.7 ± 10.6 %) or the Z-plasty (PDS 28.8 ± 19.2 %; Stratafix 44.5,35.6 %). For the non-normally distributed data, a non-parametric statistical method was applied.

### 3.4 Cyclic test

No significant differences were observed in the cyclic test between suture materials for the turndown flap (PDS 17.8 ± 4.6 %, Stratafix 19.0 ± 5.4 %) or the Z-plasty (PDS 12.9 ± 3.4 %, Stratafix 15.8 ± 6.6 %). Comparisons between the suture techniques showed no significant differences.

### 3.5 Tear-off test

For the Z-plasty, Stratafix significantly outperformed PDS in maximum force (PDS 108.5 ± 22.2N; Stratafix 142.3 ± 23.5N, p < .01) and maximum tension (PDS 5.5 ± 1.2N/mm^2^; Stratafix 7.2 ± 1.1N/mm^2^, p < .01). The Z-plasty showed significantly more maximum tension regardless of suture material (PDS p < .001; Stratafix p < .001).(see Table 1, Table 2)

**Table 1:** Direct comparison: barbed sutures with smooth monofilament PDS.

	Barbed suture (Stratafix)	smooth PDS
Tying method	knotless	equires knot
Tension distribution	even tension	localized at knot
Surgical time	reduced	longer because knot
Flexibility	limited flexibility, barbs prevent slipping	high flexibility due to individual stitches.
Repositioning	difficult to adjust	can be adjusted
Cost	generally more expensive	typically less expensive
Tissue trauma	increased from barbs	no barbs
Ease of Use	specific technique, good handling	familiar and standard technique
Healing	enhances healing through even tension distribution	reliable support for slow-healing tissues
summary	tissue damage, adjustability, running technique, limited flexibility, limited to even tension distribution	longer operation time, tension maximum at the knot site, knot placement can be difficult, knot related complications/human error

**Table 2:** Cross-section measurement in mm according to group classification.

	Group	n (Group size)	Suture technique	Suture material	Mean [mm]	SD [mm]
Native	1	10	/	/	20.9	0.6
Tendon Rupture	2	10	Turndown flap	PDS	19.5	1.0
	3	10		Stratafix	18.5	1.0
Tendon Plasty	4	10	Z-Plasty	PDS	19.8	1.2
	5	10		Stratafix	19.9	1.2

For elongation, Z-plasty with Stratafix (107.2 ± 40.4 %) was significantly higher than with PDS (62.6 ± 38.4 %, p < .001). No significant differences were observed for the turndown flap in maximum force, tension, or elongation (see Table 3 for details).

**Table 3:** Significance of the means for the biomechanical properties.

	Turndownflap		Z-plasty		native	p-values
	PDS	Stratafix	PDS	Stratafix	M ± SD	
Creep strain [%]	43.6 ± 28.1^a,b,e^	40.7 ± 10.6 %^a,c,e^	25.2 ± 22.6–59.0^b,d,e^	31.7 ± 21.6–55.1^c,d,e^	409,9 ± 157,1^e^	a>0.05; b > 0.05; c > 0.05; d > 0.05; e < 0.001
Elongation [%] (Cyclic test)	17.8 ± 4.6^a,b,e^	19 ± 5.8^a,c,e^	12.9 ± 3.4^b,d,e^	15.8 ± 6.6^c,d,e^	37.8 ± 21^e^	a>0.05; b > 0.05; c > 0.05; d > 0.05; e < 0.001
Maximum tension [N/mm^2^]	3.0 ± 0.5^a,b,e^	3.4 ± 0.8^a,c,e^	5.5 ± 1.2^b,d,e^	7.2 ± 1.1^c,d,e^	69.2 ± 14^e^	a>0.05; b < 0.001; c < 0.001; d = 0.008; e < 0.001
Maximum force [N]	52.4 ± 14.6^a,b,e^	58.9 ± 16.5^a,c,e^	108.5 ± 22.2^b,d,e^	142.3 ± 23.6^c,d,e^	1448 ± 322.4^e^	a>0.05; b < 0.001; c < 0.001; d = 0.006; e < 0.001
Maximum elongation [%]	49.3 ± 28.9^a,b,e^	53.6 ± 46.3^a,c,e^	62.6 ± 38.4^b,d,e^	107.2 ± 40.4^c,d,e^	1047 ± 375.7^e^	a>0.05; b > 0.05; c = 0.008; d = 0.029; e < 0.001

*Note.* The small superscript letters indicate the p-values for pairwise comparison.

### 3.6 Type of suture and material failure

Native tendons tore equally proximally or distally, below or above the universal testing machine clamping.

For Z-plasty with Stratafix, the tendon tissue ruptured before the actual suture failed, with PDS, suture failure preceded tissue rupture. For the turndown flap according to Silfverskiöld, material failure was observed before any tissue rupture, regardless of the material used.

## 4 Discussion

### 4.1 Key findings

This biomechanical analysis examined the two suture materials, Stratafix as a barbed knotless suture and regular PDS as a smooth monofilament suture, with two different suture techniques. One is the primary used in chronic and necleted tendon repair turndown flap according to Silfverskiöld, and one is used to lengthen tendons and various other tissues, the Z-plasty. A porcine flexor model was used due to the similarity to the human achilles tendon, since the ATR is the most common rupture.^26^^,^^28^

The cross-sectional measurement using laser optics and improved experimental design allowed to minimize the standard deviation.^25^ This indicates that all observed differences are likely due to the variation of suture material and technique rather than variations of the tendons thickness. Our results reveal no significant findings for the creep and cyclic test, regardless of the material or technique used. The tear-off test revealed significant advantages for the Stratafix suture over PDS when applied to a Z-plasty. The turndown flap could not reveal any favorable combination.

Failure analysis showed tendon tissue rupture before material failure for Z-plasty with Stratafix, for PDS on the other hand material failure precered the tissue rupture. This observation is particularly interesting when you think of material testing. Here the stratafix suture bore significantly less load than the smooth PDS. The combination of Stratafix with Z-plasty technique seems to offer greater strength and tension capacity than smooth PDS, particularly in tear-off tests. This indicates that Stratafix may be a preferred choice when performing a Z-plasty that requires high strength, potentially reducing the suture breakage rate and having the advantages of barbed sutures. When comparing the results of the Z-plasty with the turndownflap, the lack of significant differences for the turndownflap suggests that the technique does not fully utilize the even tension distribution and therefore failed under the loading conditions.

### 4.2 Explanation of rupture mechanism

With barbed sutures, the overall tension can be adjusted millimeter-wise, avoiding slipping during the first stitch. This improves the overall handling experience and material thinning.^29^ This is because knot slippage caused by suture failure is often compensated with overtightening knots and excess use of suture material. This damages the tissue, the overall repair and the healing processes.^3^^,^^29^^,^^30^ The knotless barbed suture eliminates knot-tying issues, and the barbs' even tension distribution strengthens the Z-plasty. Additionally, the difference in elongation for the Z-plasty of approximately 44 % may indicate pathological tendon lengthening or, based on our failure analysis, actual tendon tear out rather than suture failure at the knot. These findings support our hypothesis that the barbed Stratafix suture may distribute forces more evenly.

The turndownflap likely places different types of stress on the suture material, leading to comparable results across all biomechanical tests. Crucially, there was no evidence of pathological tendon elongation. This is most likely due to the fact that the Stratafix suture is similarly constructed with polydioxanone, with the key distinction being the knotless design and barbs. Failure analysis indicates that all material failures occur around the weight-bearing sutures. The barbed designs may only become apparent at higher loads or with a specific tissue-barb ratio.

### 4.3 Comparison with other research

The observed differences align with existing literature about barbed sutures. They emphasize the importance of choosing the right suture material and more importantly, the suture technique. Studies have shown that the barbed sutures and knotless design perform well when incorporating enough tissue into the repair.^3^^,^^6^^,^^30^ Our findings are consistent with this, suggesting that the Stratafix outperforms PDS under high loads in the tear-off test and revealing no significant differences in the creep and cyclic test. Further our study diverges from other studies, revealing less strength for the turndown flap; this may be attributed to slight differences in our experimental design or the tendon preparation.^25^^,^^31^^,^^32^

### 4.4 Clinical implications

Barbed sutures are ideal for high-tensile procedures like Z-plasty, offering even tension distribution and reduced knot-related failures. Clinical situations that require high flexibility like the turndownflap, smooth PDS might be more suitable. Furthermore, our results indicate that when both techniques present viable options, it is advisable to stick to the Z-plasty. In cases of tendon ruptures where one must resign to the turndown flaps, the Stratafix suture material can be used to achieve equal biomechanical results. This study contributes to a better understanding of how barbed sutures can benefit the overall repair. We propose optimizing suture material selection based on tissue quality and clinical requirements.

### 4.5 Comparison of human and porcine tendons for in vitro biomechanical studies

The human Achilles tendon is the strongest and largest tendon in the body, with a maximum force of approximately 1400 N.^33^ In comparison, porcine flexor tendons have been reported to exhibit a slightly higher load capacity of around 1800 N in the literature.^34^ In our study we found a maximum load of 1448 ± 322.4 N, aligning closely with previously reported values for porcine flexor tendons. The stiffness (N/mm) of porcine flexor tendons is very similar to that of the human Achilles tendon, making them a reliable surrogate for biomechanical studies. However, the maximum load capacity mentioned above may influence failure mechanics.^33^

Despite biomechanical similarities, anatomical differences must be considered when extrapolating findings for clinical applications. The human Achilles tendon has a twisted fiber arrangement and a higher proportion of type I collagen, contributing to its unique biomechanical properties. Additionally, the biomechanical properties of the human Achilles tendon are heavily influenced by age and activity level.^34^ These factors cannot be replicated in biomechanical in vitro studies. Porcine flexors provide a valuable in vitro model, but their translational findings are limited.^35^^,^^36^

### 4.6 Suggestions for future research

Comparable results are not necessarily to be expected for direct clinical application, as this is for the porcine flexor, not the human. Although porcine flexor tendons exhibit similar biomechanical properties to the human Achilles tendon, differences in collagen fiber orientation and cross-sectional shape may influence the direct clinical applicability of our findings. Therefore, future studies should validate these results using human cadaveric tendons to enhance translational clinical relevance and clarify the specific advantages of barbed sutures for ATR and tendon plasties. Future studies could benefit from expanding the range of suture materials and repair techniques and considering long-term biological factors.

## 5 Limitations & strengths

Biological influences such as tissue compatibility, wound healing and tendinous integration of the suture material cannot be simulated. The defect was placed on healthy tendon tissue and not on a tendon weakened by previous ruptures, surgery, or tendinopathies, as would be the case with a chronic rupture.^18^^,^^19^^,^^37^ The exact forces, especially in the early rehabilitation phase, are also very individual and cannot fully be captured by a measurement protocol, despite some studies suggesting that 30–50N is enough to simulate early active motion.^25^^,^^28^ The measurement protocol used also allows a comparison with other work of our group but not necessarily with other biomechanical studies on this topic because specimen preparation, data acquisition and data analysis are handled differently.^25^^,^^31^ This study's strengths are a well-controlled biomechanical setup and the use of a standardized testing protocol, ensuring high reproducibility and reliability. The freezing method for tendon preservation was thoroughly tested and standardized in our laboratory, allowing for consistent and precise specimen preparation. The choice of suture techniques, Z-plasty for tendon lengthening and the turndown flap for tendon repair, broaden the clinical implications of the findings. Multiple biomechanical tests (creep, cyclic, and tear-off) allow for a comprehensive evaluation of tendon stability under various conditions, especially interesting for barbed sutures. Additionally, the novel adaption of turndown flaps for tendon lengthening introduces an innovative concept that could expand surgical applications in the future.

## 6 Conclusion

Overall, the combination of the knotless Stratafix suture with the Z-plasty is particularly favorable and can increase primary stability by up to 32 %. The combination of the knotless design and the even distribution of force does indeed strengthen the overall stability, emphasizing the potential of barbed sutures, reducing the knot-associated risks.

## CRediT authorship contribution statement

**Henry V. Bürger:** Conceptualization, Methodology, Validation, Formal analysis, Investigation, Resources, Data curation, Writing – original draft, Visualization, Writing – review & editing. **Jahnke Alexander:** Conceptualization, Methodology, Validation, Resources, Data curation, Writing – review & editing, Visualization, Supervision. **Harz Torben:** Conceptualization, Software, Formal analysis, Resources, Data curation. **Carlos A. Fonseca-Ulloa:** Conceptualization, Software, Resources. **Markus Rickert:** Conceptualization, Validation, Resources, Writing – review & editing, Supervision. **Dirk Stolz:** Conceptualization, Validation, Resources, Writing – review & editing, Supervision.

## Consent to participate declaration

Consent to participate was not required for this study. It was a laboratory in vitro study and did not involve any human participants or patient interactions.

## Ethical considerations

An ethics approval was not required because porcine by-products from the slaughterhouse were used for the tendons. This was decided by the doctoral committee of the Justus Liebig University on March 16, 2023.

## Funding statement

This research did not receive any specific grant from funding agencies in the public, commercial or not-for-profit sectors.

## Declaration of competing interest

In the name of the authors of the manuscript ‘‘Evaluating barbed sutures: A porcine biomechanical comparison of z-plasty and turndown flap according to Silfverskiöld”, I declare that the material therein has not been and will not be submitted for publication elsewhere except as an abstract, authors do not have any commercial relationship that might lead to a conflict of interests, and finally, all authors were fully involved in the study and preparation of the manuscript and each of the authors has read and concurs with the content in the final manuscript.
